# Roles of Oxidative Stress, Apoptosis, PGC-1α and Mitochondrial Biogenesis in Cerebral Ischemia

**DOI:** 10.3390/ijms12107199

**Published:** 2011-10-21

**Authors:** Shang-Der Chen, Ding-I Yang, Tsu-Kung Lin, Fu-Zen Shaw, Chia-Wei Liou, Yao-Chung Chuang

**Affiliations:** 1 Department of Neurology, Kaohsiung Chang Gung Memorial Hospital and Chang Gung University College of Medicine, Kaohsiung 833, Taiwan; 2 Center for Translational Research in Biomedical Sciences, Kaohsiung Chang Gung Memorial Hospital and Chang Gung University College of Medicine, Kaohsiung 833, Taiwan; 3 Institute of Brain Science, National Yang-Ming University, Taipei 112, Taiwan; E-Mail:diyang@ym.edu.tw; 4 Department of Psychology, National Cheng Kung University, Tainan 701, Taiwan; E-Mail: fzshaw@yahoo.com.tw

**Keywords:** ischemia, oxidative stress, apoptosis, peroxisome proliferative activated receptor-γ co-activator 1α, antioxidant enzyme, mitochondrial biogenesis

## Abstract

The primary physiological function of mitochondria is to generate adenosine triphosphate through oxidative phosphorylation via the electron transport chain. Overproduction of reactive oxygen species (ROS) as byproducts generated from mitochondria have been implicated in acute brain injuries such as stroke from cerebral ischemia. It was well-documented that mitochondria-dependent apoptotic pathway involves pro- and anti-apoptotic protein binding, release of cytochrome *c*, leading ultimately to neuronal death. On the other hand, mitochondria also play a role to counteract the detrimental effects elicited by excessive oxidative stress. Recent studies have revealed that oxidative stress and the redox state of ischemic neurons are also implicated in the signaling pathway that involves peroxisome proliferative activated receptor-γ (PPARγ) co-activator 1α (PGC1-α). PGC1-α is a master regulator of ROS scavenging enzymes including manganese superoxide dismutase 2 and the uncoupling protein 2, both are mitochondrial proteins, and may contribute to neuronal survival. PGC1-α is also involved in mitochondrial biogenesis that is vital for cell survival. Experimental evidence supports the roles of mitochondrial dysfunction and oxidative stress as determinants of neuronal death as well as endogenous protective mechanisms after stroke. This review aims to summarize the current knowledge focusing on the molecular mechanisms underlying cerebral ischemia involving ROS, mitochondrial dysfunction, apoptosis, mitochondrial proteins capable of ROS scavenging, and mitochondrial biogenesis.

## 1. Introduction

Mitochondria are ubiquitous intracellular organelles enclosed by a double membrane structure. The primary function of mitochondria is the production of cellular energy in the form of adenosine triphosphate (ATP) by the mitochondrial respiratory chain through oxidative phosphorylation. Mitochondrial oxidative phosphorylation consists of five multienzyme complexes (Complexes I–V) located in the mitochondrial inner membrane [1]. Biochemical evidence suggested that the majority of cerebral ATP consumption is used for operation of the electrogenic activity of neurons [2]. Adequate energy supply by mitochondria is therefore essential for neuronal excitability and survival. In addition to energy production, mitochondria also play a crucial role in the generation of reactive oxygen species (ROS) and regulation of apoptosis [3,4], both have been implicated as important factors in the pathogenesis of neurodegenerative diseases and cerebral ischemia [3,5].

Stroke is the leading cause of adult disability and the second or third leading cause of death in most of the developed countries. Data from the World Health Organization suggest that annually 15 million people worldwide suffer a stroke. Of these, 5 million die and another 5 million are left permanently disabled, placing an enormously heavy burden on family and society (The Atlas of Heart Disease and Stroke 2011 from http://www.who.int/cardiovascular_diseases/resources/atlas/en/). The majority of patients with stroke experience thrombotic or embolic events that result in ischemic brain injury. Thus, the major aim of stroke research is to develop therapeutic interventions to reduce brain damage through the understanding of pathogenic molecular mechanisms underlying ischemic insults.

Recent evidence has suggested an intimate link between an excessive generation of ROS and the development of neuronal death in diverse neurological disorders. These include chronic neurodegenerative diseases such as amyotrophic lateral sclerosis and epilepsy as well as acute brain injury like brain trauma and cerebral ischemia [6,7]. It was also reported that oxidative stress from cerebral ischemia may promote amyloid β production which links the potential connection between stroke and Alzheimer’s disease [8,9]. Excessive ROS generation can induce the functional and structural damage of neuronal cells and may play an important role in the pathophysiology of cerebral ischemia [10–12]. As mitochondrial dysfunction with excessive oxidative stress may play a crucial role in ischemic cascades, counteracting this detrimental effect through better understanding the neuronal damage resulting from apoptotic or necrotic cell death should have the perspective in treating ROS-related disorders such as ischemic stroke.

Emerging evidence showed that ROS-detoxifying system and mitochondrial biogenesis may play a pivotal role as endogenous protective mechanisms in neurodegenerative diseases and cerebral ischemia [12–15]. This review will focus on mitochondrial dysfunction in cerebral ischemia-associated neuronal death and the potential role of peroxisome proliferator-activated receptor-γ (PPARγ) co-activator 1α (PGC1-α) on ROS and mitochondrial biogenesis.

## 2. Ischemic Cascade Involving Mitochondria and ROS

**Figure 1:**
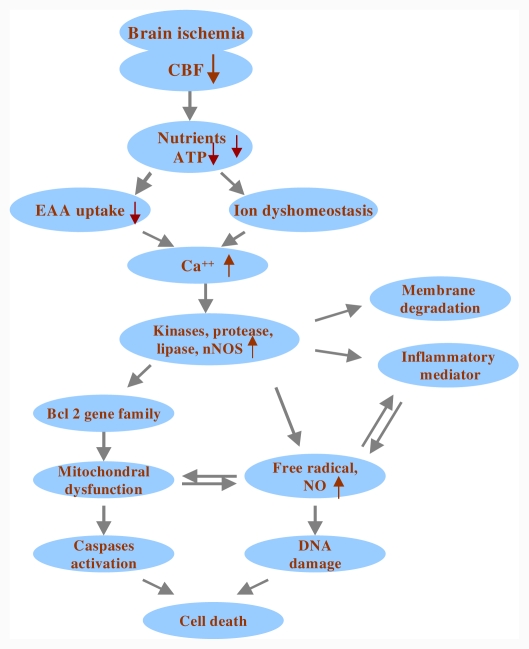
Schematic overview of selected cellular events in the ischemic brain. The ischemic event begins with reduced blood flow to the area supplied by the occluded arteries. The lack of oxygen, glucose, and other nutrients leads to an ischemic cascade culminating in cell death. **EAA** = excitatory amino acid; **ATP** = adenosine triphosphate; **CBF** = cerebral blood flow; **nNOS** = neuronal nitric oxide synthase; **NO** = nitric oxide; ↑ and ↓ denote increase and decrease, respectively.

The pathophysiological cascades following cerebral ischemia have been characterized in animal models of stroke [16–18]. The ischemic event begins with reduced blood flow to the areas supplied by the occluded arteries. The lack of oxygen, glucose, and other nutrients leads to a state of disturbed cellular homeostasis, culminating in cell death. The cellular events leading to ischemic neuronal death have been extensively studied and the probable sequence of events involved in ischemic neuronal death is depicted in Figure 1. In this section, we focus on the detrimental effects of oxidative and nitrosative stress generated from mitochondria over neuronal injury under cerebral ischemia.

### 2.1. Mitochondria and Oxidative Stress in Cerebral Ischemia

Oxidative stress is defined as an imbalance between the production of ROS and the ability to readily detoxify the reactive intermediates in a biological system. The effects of oxidative stress depend upon the severity of these changes. A small perturbation may be overcome by the endogenous anti-oxidant system. However, severe oxidative stress can cause cell death either via an apoptotic or a necrotic pathway [19]. In living cells including neurons, ROS can be generated under stimuli such as hypoxia, serum deprivation or cytokine stimulation by several sources mainly including NADPH oxidase, 5-lipoxygenase and mitochondria [20]. Among them, mitochondria are the major organelles that produce ROS within cells [3,21,22]. Under successive oxidative stress the free electrons on the mitochondrial electron transport chain may leak out and react with molecular oxygen, thereby generating superoxide anion as metabolic byproducts during respiration. Nitric oxide (NO) may react with superoxide anion to produces the highly reactive peroxynitrite anion (ONOO^−^) that reacts with DNA, proteins, and lipids. Together, modification of these macromolecules by ROS and/or reactive nitrosative species (RNS) plays an important role in many physiological and pathological conditions, notably ischemia-reperfusion injury, neurodegenerative diseases, aging, and cancer [12,23–25]. Thus, it is crucial to maintain low levels of ROS for normal cell functions, whereas prolonged increases in mitochondrial activity may carry an inherent risk of increasing ROS levels. After cerebral ischemia, the balance between ROS production and clearance are compromised, which may result in oxidative stress-induced signaling and cell injury. The pathogenic role of oxygen free radicals in ischemic brain injury has been reviewed elsewhere [3].

### 2.2. Mitochondria and Nitrosative Stress in Cerebral Ischemia

Brain ischemia also triggers the activation of various isoforms of nitric oxide synthases (NOSs). These include the constitutively expressed neuronal (nNOS) and endothelial (eNOS) and the inducible (iNOS) isoforms. The role of NO in ischemic brain injury is complex. Excessive NO formation may be cytotoxic by directly inhibiting enzymes that catalyze vital cellular functions involved in energy metabolism and DNA synthesis. The deleterious effects of NO might be attributed to its well-known affinity for iron and thiol groups [26,27]. NO may also contribute to free radical generation by forming peroxynitrite anions, which then forms the cytotoxic hydroxyl radical and superoxide anion [28].

It has been shown that mitochondria contain their own isoform of NOS, mitochondrial NOS (mtNOS), at the inner membrane [29,30]. The existence of mtNOS is still under debate because of the concerns for contamination during mitochondrial preparations and the lack of antibody specificity for mtNOS as these antibodies also cross-react with other NOS isoform, including eNOS and nNOS [31]. Nevertheless, more recent data support mtNOS as an independent form of NOS and mtNOS activities were reported in multiple organs from from mouse and rat, including brain, heart, kidney, thymus, and skeletal muscle [32]. mtNOS is thought to be synthesized in the cytosol and then translocated into mitochondria [33] but the mechanism of this translocation remains unknown at present. As mtNOS continuously controls mitochondrial respiration [29,30], it is considered a key enzyme of reperfusion injury [34] and may be related to apoptosis after stroke. To date, only a limited number of studies has reported correlation between mtNOS and ischemia [35]. Uncovering the roles of mtNOS after cerebral ischemia may thus provide novel therapeutic strategies for stroke patients.

Recent studies [36] also suggested that nitrosative stress due to generation of excessive NO could mediate excitotoxicity in part by triggering protein misfolding, aggregation, and mitochondrial fragmentation. S-Nitrosylation, the covalent reaction of NO with specific protein thiol groups, represents a convergent signal pathway contributing to NO-induced protein misfolding and aggregation that compromised dynamics of mitochondrial fission-fusion process, thus leading to neurotoxicity [36]. Role of nitrosative stress and mitochondrial dysfunction in cerebral ischemia is less well explored and warrants further studies.

## 3. Pro- and Anti-Apoptotic Proteins Associated with Mitochondria-Dependent Apoptosis in Cerebral Ischemia

Mitochondria and neuronal death in ischemia-induced cell death is no exception to the emerging complexities of the molecular control of other neurological disorders such as neurodegeneration or seizure [13,37]. Cell death in the ischemic brain reflects a transition between activated pro-death factors and cellular pro-survival responses over hours or even days. Neurons in the final stages of their demise often present a mixed picture with cell death in a programmed/controlled (apoptotic) or uncontrolled/passive (necrotic) manner.

Programmed cell death mechanisms associated with cellular apoptosis have been shown in human and animal studies that support apoptotic cell death playing an important role in ischemia-induced brain damages [38–41]. Factors such as variation in duration and severity of ischemia, metabolic disturbance, bioenergetics failure during or after ischemia and age- or genetic-specific factors may all contribute to determining the eventual pathway of cell death [42,43]. A critical determinant of the eventual cell death fate resides in intracellular ATP concentration, the production of which depends on the structural and functional integrity of the mitochondria. Whereas ATP depletion is associated with necrosis, ATP is required for the progression of apoptosis [44].

In ischemic stroke, a necrotic core is surrounded by a zone known as the “ischemic penumbra” that is less affected and functionally silent tissue. Penumbral area, resulted from partial reduction of cerebral blood flow, remains metabolically active and represents the region where opportunities exist for salvage of neurons via post-stroke therapy [45]. Recent research has revealed that many neurons in the ischemic penumbra or peri-infarct zone may undergo apoptosis after several hours or days [46–48] that can potentially be rescued after stroke. Intervention of this apoptotic process in the penumbra would seem to be an achievable therapeutic target for limiting cerebral infarct volume after clinical stroke. To update our knowledge about apoptosis in this ischemic paradigm would provide a basis for the novel anti-apoptotic intervention.

Given its role as the cellular powerhouse, the mitochondrion is emerging as a key participant in cell death because of its association with an ever-growing list of apoptosis-related proteins [49,50]. The evidence of Bcl-2 family involved in ischemia-induced neuronal cell death has also been demonstrated [51–55]. The Bcl-2 protein family is a principal regulator of mitochondrial membrane integrity and function. According to the structural homology they are classified as the anti-apoptotic proteins including Bcl-2, Bcl-xL, and Bcl-w, the pro-apoptotic proteins including Bax and Bak, as well as the BH3-only proteins including Bad, Bid, Bim, Noxa, and p53-upregulated modulator of apoptosis (PUMA) [3].

A variety of key events in apoptosis focus on mitochondria, including the release of several apoptogenic factors (such as cytochrome *c*, apoptosis-inducing factor or AIF, endonuclease G, Smac/DIABLO, and HtrA2/OMI), changes in electron transport, loss of mitochondrial transmembrane potential, altered cellular redox states, and participation of pro- and anti-apoptotic Bcl-2 family proteins [49,56]. Studies have shown that after cerebral ischemia, BH3-only proteins were upregulated, denoting that this ischemic process triggers various apoptotic pathways [57–60]. One of the decisive steps of the apoptotic cascade is related to the mitochondrial permeability transition pores (mPTPs) [61]. Transient opening of mPTPs in the mitochondrial inner membrane under conditions of cellular stress causes the mitochondrial transmembrane potential to collapse and elicits the release of cytochrome *c* as well as other pro-apoptotic molecules that together initiate the apoptotic cascade. Cytochrome *c* interacts with apoptosis activating factor-1 (Apaf-1), deoxyadenosine triphosphate (dATP), and procaspase-9 to form the apoptosome, which activates procaspase-9 and following with caspase-9 to cleave and activate caspase-3 [62–64]. Smac released from mitochondria binds to, and hence neutralizes, the anti-apoptotic effects of the X chromosome-linked inhibitor-of-apoptosis protein (XIAP), which can prevent procaspase activation and inhibits activities of activated caspases after cerebral ischemia [65,66]. AIF also translocates from mitochondria to the nucleus and induces apoptosis after focal cerebral ischemia and inhibition of poly(ADP-ribose) polymerase and Bid reduces nuclear AIF translocation [67].

## 4. PGC-1α–an Endogenous Protective Mechanism Involving ROS and Mitochondria Biogenesis in Cerebral Ischemia

Under cerebral ischemia, a detrimental pathway begins with decreased CBF, increased ROS, triggering apoptotic pathway, and finally leads to neuronal death [10,16]. On the other hand, there are molecules that can prevent caspase activation in the cytosol. Inhibitor of apoptosis protein (IAP) family is known to suppress apoptosis by preventing activation of procaspases and inhibiting enzymatic activity of active caspases [68] or interacting with Smac [69]. The PI3-K/Akt survival signaling pathway is upregulated by superoxide dismutase 1 overexpression and suppress ischemic neuronal death during stroke [70].

Previous studies have shown that the PGC-1α is a potent stimulators of mitochondrial respiration and gene transcription in liver, heart, and skeletal muscle [71]. Several neurodegenerative diseases such as Parkinson’s disease, Alzheimer’s disease, and Huntington’s disease have been associated with impaired mitochondrial function and decreased expression of genes involved in mitochondrial oxidative phosphorylation [72]. It was reported that PGC-1α knockout mice have a striking spongiform lesion in the striatum, the brain region primarily affected in Huntington’s disease patients or lesions observed in substantia nigra and hippocampus, two regions severely affected in patients suffering from Parkinson’s disease and Alzheimer’s disease, respectively [73]. Activation or overexpression of the PGC-1α could be used to compensate for neuronal mitochondrial loss and suggest that therapeutic agents activating PGC-1α would be valuable for treating neurodegenerative diseases in which mitochondrial dysfunction and oxidative damage play an important pathogenic role [74]. Recent studies have revealed that oxidative stress and the redox state of ischemic neurons are also implicated in the signaling pathway that involves PGC1-α. Two mitochondrial proteins-uncoupling protein 2 (UCP2) and superoxide dismutase 2 (SOD2) that are both regulated by PGC1-α play a pivotal role to counteract the damaging effect elicited by excessive oxidative stress [13]. In this section, we review the importance of PGC-1α-mediated ROS metabolism and mitochondrial biogenesis in relation to cerebral ischemia.

### 4.1. PGC-1α in Mitochondria-Related ROS Metabolism under Cerebral Ischemia

Peroxisome proliferator-activated receptors (PPARs) are ligand-activated transcription factors that may regulate lipid and lipoprotein metabolism, glucose homeostasis, cell proliferation and differentiation, as well as apoptosis.

Importantly, PPARs also modulate the inflammatory and oxidative responses [75]. Evidence revealed that PPARs have beneficial effects in inflammatory diseases through regulation of cytokine production and adhesion molecule expression by interfering with the transactivation capacity for nuclear factor-κB (NF-κB), activator protein-1 (AP-1), and signal transducers and activators of transcription (STAT) [75–77]. It is well documented that activation of PPARγ can attenuate post-ischemic inflammation and damage [21,78,79]. In recent studies, PPAR delta also revealed its pivotal role in ischemic injury and warrants further investigation for the development of therapeutic strategy for stroke [80,81]. Since the identification of PPARγ as a PGC-1α transcription factor target, a variety of additional PGC-1 target nuclear receptors have been identified which include PPARα, PPARβ/delta, thyroid hormone receptor, retinoid receptors, glucocorticoid receptor, estrogen receptor, liver X receptor, and the estrogen-related receptors [82]. PGC-1α is a transcriptional coactivator that transduces many physiological stimuli into specific metabolic programs such as gluconeogenesis, thermogenesis, fatty acid oxidation and mitochondrial biogenesis [82–84]. Consistent with its emerging role as a central regulator of energy metabolism, PGC-1α is abundantly expressed in tissues with high metabolic rates such as in striated muscle, brown adipose tissue, liver, and brains [13,82].

PGC-1α is activated under oxidative stress. It has been reported that, in cultured skeletal myotubes with ischemia-like conditions, PGC-1α gene expression is induced [85]. PGC-1α is also expressed in the mouse cerebral subcortex under hypobaric hypoxia [86] and in skeletal muscle with hibernation, a known hypoxic state [87]. These studies denote the pivotal roles of PGC-1α in tissues under ischemia-hypoxia condition. In neuronal cells, it was reported that PGC-1α is required for the induction of many ROS-detoxifying proteins, including glutathione peroxidase, catalase, UCP2 and SOD2 [13]. Down-regulation of PGC-1α expression in mice exacerbates the detrimental effects of 1-methyl-4-phenyl-1,2,3,6-tetrahydropyridine (MPTP) to substantia nigra or kainic acid to hippocampus [13]. In contrast, an increased PGC-1α expression may protect cultured neural cells from oxidative stress-mediated cell death [13]. Nevertheless, the exact roles of PGC-1α in ROS metabolism under cerebral ischemia remain largely unexplored. In view of the crucial relationship between ischemia-induced neuronal damage and overproduction of ROS, it is reasonable to speculate the importance of PGC-1α in this paradigm.

Two important ROS-detoxifying proteins have been identified in mitochondria, namely UCP2 and SOD2 [88,89]. They play a crucial role in the fate of neurons and damage progression after ischemic stroke by regulation of ROS production [90,91]. Upregulation of UCP2 after cerebral ischemia decreased the release of ROS and reduced neuronal loss in the brain tissue that offers a novel neuroprotection against ischemic brain injury [21,91,92]. Furthermore, animals overexpressing SOD2 showed a protective effect against oxidative stress-induced neuronal damage after transient focal cerebral ischemia [93,94]. Importantly, UCP2 and SOD2 have been proposed to be direct targets downstream of PGC-1α [13,95]. We have demonstrated recently that under transient ischemic condition, ROS overproduction may stimulate the activation of PGC-1α signaling pathway, further triggering upregulation of UCP2 and SOD2 in hippocampal CA1 neurons [12]. Knock-down of PGC-1α expression by pretreatment with PGC-1α antisense oligodeoxynucleotide (ODN) diminished the expression of UCP2 and SOD2 that led to exacerbation of oxidative damage and augmentation of delayed neuronal death in the hippocampus after transient global ischemia. Therefore, PGC-1α signaling pathway may emerge as a new potential target for future development of more effective neuroprotective strategies against ischemic brain injury.

### 4.2. PGC-1α Signaling Pathway and Mitochondrial Biogenesis under Cerebral Ischemia

Mitochondria are important for cellular homeostasis. Recent studies have shown that mitochondrially formed oxidants are mediators of molecular signaling and have been implicated in mitochondria-dependent apoptosis. However, oxidative stress and the redox state are also involved in the survival signaling pathway of the stressed cells. Nevertheless, mitochondria are not static organelles. Fluctuating homeostatic demands and inherent production of ROS cause progressive damage of mitochondria and require dynamic regulation of their turnover, cellular contents, biological functions, as well as total numbers of this vital intracellular organelle [96]. This is in particular crucial for proper function of post-mitotic neurons; however, the information is limited regarding the role of mitochondrial biogenesis in neural cells. Mitochondrial abundance as well as the copy number and integrity of mtDNA in mammalian cells may alter under pathological conditions with increased oxidative stress and during aging process [97,98]. Within a certain level, ROS may activate stress responses by altering expression of specific nuclear genes to maintain the normal energy metabolism and cope with hazardous environments for cell survival. Once beyond a certain threshold, ROS may cause oxidative damage to mtDNA and other biomolecules of the affected cells to elicit apoptosis. It has been demonstrated that a number of protein factors encoded by nuclear genes are involved in the biogenesis of mitochondria and respiratory functions [98]. Nuclear respiratory factors 1 and 2 (NRF-1 and NRF-2) are transcriptional regulators that act on the nuclear genes coding for constituent subunits of the oxidative phosphorylation system. In addition, they also regulate the expression of many other genes involved in mtDNA replication via binding to the consensus sequences in the promoters of the oxidative phosphorylation genes in the nucleus [97–99]. Mitochondrial transcription factor A (Tfam) is a transcription factor that acts on the promoters within the D-loop region of mtDNA and regulates the replication and transcription of the mitochondrial genome [98]. It has been established that the Tfam gene contains consensus-binding sites for both NRF-1 and NRF-2, which provide a unique mechanism for the cells to integrate the expression of nuclear DNA-encoded proteins with the transcription of genes encoded by mtDNA [97,98].

**Figure 2:**
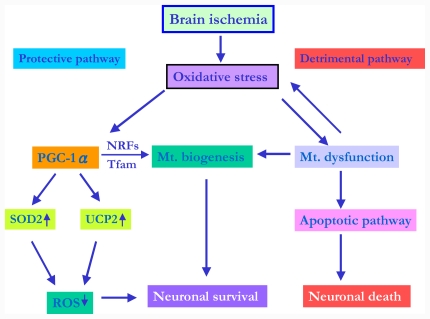
Proposed role of PGC-1α in ischemic condition. ROS overproduction may stimulate the activation of PGC-1α signaling pathway, further triggering upregulation of mitochondrial proteins, including UCP2 and SOD2, in ischemic neurons. PGC-1α also regulates NRF-1, NRF-2 and Tfam expression as well as mitochondrial biogenesis that may have protective effects in ischemic condition. **PGC-1α** = peroxisome proliferative activated receptor-γ co-activator 1α; **UCP2** = uncoupling protein 2; **SOD2** = superoxide dismutase 2; **NRFs** = Nuclear respiratory factors; **Tfam** = Mitochondrial transcription factor A; ↑ and ↘ denote increase and decrease, respectively.

It has been shown that PGC-1α may be a major regulator of mitochondrial biogenesis [84]. A recent study demonstrates that hypoxia-ischemia induces mitochondrial biogenesis. After hypoxia, increases in mitochondrial DNA, total mitochondrial number, expression of the mitochondrial transcription factors downstream of PGC-1α (NRF1 and Tfam), and the mitochondrial protein HSP60 are detected [15]. In view of mitochondria as an energy center and important for cellular homeostasis, exploring the roles of mitochondrial biogenesis as an endogenous protective response to cope with ischemic insult may help us to develop a strategy to enhance this beneficial effect and counteract the ischemia-related detrimental effects. In our recent study, we showed that, under transient global ischemia, the PGC-1α signaling pathway is activated, which may trigger the UCP2 and SOD2 expression and promote mitochondrial biogenesis in the hippocampal CA1 subfield. In keeping with the role of mitochondrial biogenesis as a potential endogenous protective mechanism [15,100], boosting the signal transduction pathways upstream of mitochondrial biogenesis, such as the PGC-1α signaling cascade, may therefore become a novel target for a therapeutic strategy against ischemic brain damage [14]. The probable roles of PGC-1α with mitochondria proteins-UCP2 and SOD2-as well as mitochondrial biogenesis in ischemic condition is depicted in Figure 2.

## 5. Conclusions

Numerous studies report the involvement of ROS in cell death after cerebral ischemia. ROS contribute not only to injury of macromolecules such as lipids, proteins, and DNA, but also to transduction of apoptotic signals. Mitochondrial dysfunctions occur as a consequence of cerebral ischemia and promote ischemia-induced neuronal cell death, especially the intrinsic pathway of apoptotic cell death. Conversely, endogenous protective pathways exist to counteract these detrimental effects induced by ischemia including mitochondria proteins UCP2 and SOD2, which are all regulated by PGC-1α. Therefore, mitochondria can be considered as a target for potential neuroprotective strategies in cerebral ischemia. Future studies of these cell death/survival mechanisms subsequent to ischemic attack may provide unique information regarding molecular targets for therapeutic strategies in clinical stroke. Protection of the mitochondria from bioenergetics failure and oxidative/nitrosative stress resulting in apoptosis in the ischemic tissue may open a new vista to the development of more effective neuroprotective strategies against ischemia-induced brain damage.
